# A new non-invasive approach based on polyhexamethylene biguanide increases the regression rate of HPV infection

**DOI:** 10.1186/1472-6890-12-17

**Published:** 2012-09-25

**Authors:** Antonio Gentile, Sandro Gerli, Gian Carlo Di Renzo

**Affiliations:** 1 Family Planning Clinic Terme Vigliatore, A.S.P., 5 Via Nazionale S. Biagio, 154 98050 Messina, Italy; 2 Department of Obstetrics and Gynecology, University of Perugia, Perugia, Italy

**Keywords:** HPV, Polyhexamethylene biguanide, Intraepithelial lesions, Regression rate

## Abstract

**Background:**

HPV infection is a worldwide problem strictly linked to the development of cervical cancer. Persistence of the infection is one of the main factors responsible for the invasive progression and women diagnosed with intraepithelial squamous lesions are referred for further assessment and surgical treatments which are prone to complications. Despite this, there are several reports on the spontaneous regression of the infection.

This study was carried out to evaluate the effectiveness of a long term polyhexamethylene biguanide (PHMB)-based local treatment in improving the viral clearance, reducing the time exposure to the infection and avoiding the complications associated with the invasive treatments currently available.

**Method:**

100 women diagnosed with HPV infection were randomly assigned to receive six months of treatment with a PHMB-based gynecological solution (Monogin®, Lo.Li. Pharma, Rome - Italy) or to remain untreated for the same period of time.

**Results:**

A greater number of patients, who received the treatment were cleared of the infection at the two time points of the study (three and six months) compared to that of the control group. A significant difference in the regression rate (90% Monogin group *vs* 70% control group) was observed at the end of the study highlighting the time-dependent ability of PHMB to interact with the infection progression.

**Conclusions:**

The topic treatment with PHMB is a preliminary safe and promising approach for patients with detected HPV infection increasing the chance of clearance and avoiding the use of invasive treatments when not strictly necessary.

**Trial registration:**

ClinicalTrials.gov Identifier NCT01571141

## Background

Human papilloma virus (HPV) is a worldwide genital infection and it is considered the most common cause of sexual transmitted infections (STIs). It is possible to count more than 100 HPV types known to infect humans and among these 13 have been classified as “high-risk” (HR) by the WHO International Agency for Research on Cancer. In particular, types 16 and 18 have been linked to more than 70% of cervical cancer [1, 2].

Currently, the only tool that medicine has to identify the HPV infection early is cervical cancer cytological screening and women diagnosed with high-grade intraepithelial squamous lesions (H-SIL) are referred for further assessment with colposcopy and biopsy to prevent the invasive progression.

On the other hand, a follow up check is carried out on women with detected low-grade intraepithelial squamous lesions (L-SIL) due the increased risk of developing H-SIL [3, 4].

HPV transmission depends on several factors such as the infectivity of the organisms, sexual behavior and the effectiveness of control interventions.

The majority of the treatments currently available (cytotoxic, physically ablative, excisional, and immunomodulatory therapies for genital warts) may reduce the symptoms but are relatively ineffective in eradicating the infection and/or decreasing the likelihood of disease transmission [5].

So far, the surgical removal of the lesions followed by the local application of therapeutic agents is considered the most effective therapy, avoiding the progression to pre-cancer lesions which are more likely to occur when the infection persists over time. Despite this, all the invasive treatments currently performed (cryotherapy, surgical excision, electrosurgery or laser vaporization with CO_2_) are associated with several complications including cervical stenosis, bleeding and infection. Furthermore, there are a few reports on the spontaneous regression of low- and medium-grade lesions which offer the possibility to avoid the treatment [6]. In order to take full advantage of it and to increase the regression rate, the identification of a non-invasive therapeutic agent able to interact with the HPV infection is strongly warranted.

The clinical application of polyhexamethylene biguanide (PHMB)-based compounds has been mainly reported for the treatment of bacterial vaginosis and wounds. PHMB is a strong base which interacts with acidic phospholipids leading to increased fluidity and permeability of the bacterial cellular membrane and consequently to the death of the pathogen. Neutral phospholipids are not affected by PHMB thus justifying the low toxicity against the human cells [7]. Furthermore, the antiviral activity of PHMB and other biguanides has been also reported [8, 9].

In this study, a long-term treatment with a PHMB-based gynecological solution (Monogin®, Lo.Li. Pharma, Rome - Italy) has been performed on patients diagnosed with HPV infection to evaluate the effectiveness in increasing the spontaneous regression rate.

## Methods

From January to December 2010, 100 women were enrolled at the “Consultorio Familiare Terme Vigliatore, A.S.P. 5” of Messina, Italy. Subjects aged between 30 and 45 years and diagnosed with the HPV infection after the pathologic Pap smear (ASC-H, L-SIL and H-SIL), a positive “high risk” HPV DNA test and a positive colposcopy examination (abnormal transformation grade 1 or 2) were included in the study. Exclusion criteria were wart therapy in the previous six months, pregnancy, invasive disease, immunosuppression and previous HPV vaccination. All the patients gave their written informed consent before entering the study which was approved by the SIFIOG (Italian Society of Phototherapy and Dietary Supplements in Obstetrics and Gynecology) ethical committee.

### Protocol

This hyphotesis-generating study was performed to investigate whether a long treatment with a PHMB based gynecological solution, which has been reported to have biocidal activity, may improve the regression rate of HPV infection.

A pre-enrolment screening questionnaire was used to record the social and medical history of the patients and no differences were observed. Patients were randomly assigned to two groups using a computer based program.

Group A consisted of 50 women who received treatment with a polyhexamethylenbiguanide (PHMB) gynecological solution (Monogin®, Lo.Li. Pharma, Rome - Italy) every three days for fifteen days and then every fifteen days for the subsequent six months. Monogin® is a mono-dose gynecological liquid-gel constituted by PHMB, EDTA, glycerol, hydroxymethyl cellulose, potassium chloride, lactic acid and water; the administration did not require any particular procedure and it was self-administered by the patients before bedtime, following the information reported in the leaflet.

Group B, consisting of other 50 women, did not receive any treatment and was considered as control group.

Evaluation of PHMB effectiveness in increasing the regression rate of the infection was performed by taking cervical scrapings for HPV analysis, Pap smears and a colposcopy examination. This procedure was performed at the enrollment and repeated after three (T1) and six months (T2), following the initial diagnosis of HPV infection.

### HPV analysis

HPV detection was performed using the HPV high-risk test (HC2, Qiagen, Baltimore, USA) which detects 13 high risk oncogenic HPV types (16, 18, 31, 33, 35, 39, 45, 51, 52, 56, 58, 59 and 68). An HPV sample was considered positive according to the Food Drug Administration (FDA) guidelines and the standard 1.0 pg HPVDNA ml^-1^ threshold was adopted.

Cervical scrape samples were collected in ThinPrep vials containing PreservCyt (Cytyc Corp., Marlborough, USA) transport medium or in Specimens Transport Medium (STM) (DNAPAP cervical sampler, Qiagen, Gaithersburg, USA) and the results were classified following the 2001 Bethesda System [10].

Colposcopy examination was performed following the general guidelines described elsewhere [11].

### Statistical analysis

To compare the regression rate of HPV infection in the two groups the Fischer’s exact test has been used and *P* values less than 0.05 were considered statistically significant.

The analysis has been conducted using GraphPad Prism software (Graph Pad Software, Inc., La Jolla USA).

## Results

All the enrolled patients completed the study. The evaluation of Pap smear, HPV DNA test and colposcopy were repeated after three months and at the end of the study and the infection was considered cleared when all the features were found negative.

Results have shown that after three months (T1) a regression rate of the infection was observed in the 66% (33/50 patients) of the patients treated with Monogin® gynaecological solution compared to the 56% (28/50 patients) of the control group (Table 1). The difference between the two groups did not reach statistical difference (RR 1.18, CI95 0.86-1.62). At the end of the study (T2) a reduction of the number of HPV positive patients was still observed in both groups with a significant increment of the regression rate in the patients who received the treatment (90% Monogin® *vs* 70% control group, *P* value = 0.023; RR 1.29, CI95 1.05-1.58; Table 2). All the treated patients were asked about adverse effects and no side effects related to the long term use of the PHMB-based gynaecological solution (Monogin®) were reported.

**Table 1:** Number of patients with or without HPV infection at T1

Group (n =50)	T1		
	Infection free	Infection	RR (CI95)
**Monogin®**	33	17	1.18 (0.86-1.62)
**Control**	28	22

**Table 2:** Number of patients with or without HPV infection at T2

Group (n =50)	T2		
	Infection free	Infection	RR (CI95)
**Monogin®**	45*	5	1.29 (1.05-1.58)
**Control**	35	15	

*P value = 0.023, respect to the control group at the T2.

At the end of the study, due to the persistence of positive colposcopy and positive Pap smear and HPV DNA test, five patients in the Monogin group and fifteen in control group were successively treated according to the national guidelines.

## Discussion

In this randomized trial we have shown that six months treatment with a PHMB-based gynaecological solution (Monogin®, Lo.Li. Pharma, Rome - Italy) increases the regression rate of the HPV infection. In particular, the treatment already exerts beneficial effects at three months compared to an untreated control group.

The presence of cervical HPV DNA is often associated with cytological and histologic changes of cervical intraepithelial neoplasia (CIN) [12]. Despite this, there are a few reports showing a spontaneous regression of a number of low- and medium-grade lesions and the majority of women clear the virus or suppress it to levels not associated with significant cervical dysplasia. Gloria Ho et al. reported that the median duration of HPV infection is 8 months and that 70% of women clear the infection after 12 months [13].

On the other hand, since the time exposure with high risk HPV types is directly connected with the risk of development of cervical cancer, the development of non-invasive therapeutic agents able to improve the viral clearance is necessary.

Up to now, only a few therapeutic agents able to interact with the infective process are known and currently prescribed. In particular, Imiquimod (Aldara®) which entered in the market in 1997, is an immune response modifier which induces the activation of congenital and acquired T-cell immunity stimulating the production of host interferon and other endogenous cytokines with antiviral properties. Another therapy currently available is represented by interferon (IFN) which are endogenous intracellular proteins possessing not only anti-cancer, but also antiviral immunomodulating effects [13]. Nevertheless, several side effects and complications are associated with the administration of these agents and the search for new safe and effective anti-HPV compounds is still warranted.

PHMB is a well-known antimicrobial agent and a number of scientific evidences on its antiviral activity have also been reported. Specific activity against cell-free viruses may be attributable to interactions between PHMB and components of the virion membrane. It has been suggested that PHMB is effective against HIV-1 in-vitro since the cellular infection is mediated by components in both the cellular membrane and the viral envelope. These interactions cause a decrease of the virulence disrupting the integrity of the viral particle [14].

Further studies confirmed the antiviral activity of biguanide-based molecules suggesting a specific mechanism that has a co-receptor dependency [9]. Additional studies also reported the in-vitro activity against HSV-1, HSV-2 and VZV at the PHMB concentration of 0.01% [8].

Marelli et al. first investigated the clinical effectiveness of a PHMB-based cream in the treatment of genital warts and the same authors suggested that PHMB can be considered an effective therapeutic option in controlling HPV infection [15]. Our study further supports these preliminary results with new clinical data. PHMB is structurally similar to naturally occurring antimicrobial peptides (AMPs) that unlike the majority of conventional antibiotics have the ability to enhance the immune response by functioning as immunomodulators [16]. Furthermore, PHMB anti-inflammatory activity has been reported suggesting that the local action of PHMB is probably due to the ability to inhibit the formation of reactive oxygen species in vitro [17].

Additionally, several cell components such as integrin α6β1, integrin α6β4 and glycosaminoglycans (GAGs) have been proposed as primary receptors of HPV in the cellular adhesion process [18, 19]. Both integrins contain binding sites for divalent cations (Mg^2+^ and Ca^2+^) which are necessary for the adhesive process [20, 21]. Furthermore, among the components of the Monogin® (PHMB, EDTA, glycerol, hydroxyethyl cellulose, potassium chloride, lactic acid and water), EDTA is a strong chelating agent which assists and enhances the action of PHMB which interacts with the negatively charged sites displacing the metallic cations essential to the integrity of the cell outer membrane [22]. The synergistic action of PHMB and EDTA may impair the viral adhesion process and prevents the spread of infection [23].

In has been reported that in the in vivo infection process the first HPV binding site is represented by the basal membrane (BM) through an heparan sulphate proteoglycan (HSPG)-dependent binding mechanism. HSPG is a linear, highly charged, anionic polysaccharide which belongs to the GAG family whom interaction with macromolecules appears to be based mainly on its anionic properties [24]. Because of this, it can be speculated an additional site of action of PHMB which impairs the HSPG-HPV binding in the BM counteracting the viral infection process. However, the mechanism of action of PHMB on viral adhesion is still not fully understood and further studies are needed to clarify it.

We speculate that on the basis of combined antimicrobial, protective, immunomodulatory, anti-inflammatory and chelating effects, PHMB and EDTA interact with HPV increasing the chance to clear the infection. In our study, this regression has been reported after three months of treatment in 66% of patients compared to the 56% in the untreated control group. The difference between the two groups became significant after six months suggesting a promising effect of the treatment in the reduction of the infection persistence overtime.

The spontaneous regression rate observed in the control group is in line to the data reported in several clinical studies [13].

On the basis of these preliminary results, further studies to evaluate the effects of PHMB both on cervical and on other types of HPV infected lesions such as common warts in the skin, oral squamous cell carcinoma and verrucous carcinoma are necessary and could provide additional information on the efficacy of this molecule in reducing the viral adhesion process.

## Conclusion

In conclusion, the results obtained from this pilot study indicate that the long term local treatment with PHMB may represent a safe and effective approach for patients with low- and medium-grade lesions enhancing the regression rate of HPV infection. In particular, this study suggests that a PHMB-based therapy may represent an alternative preliminary treatment for patients with detected HPV infection, increasing the chance to clear the infection, avoiding the complications associated with the conventional invasive treatments and reducing the time exposure to HP. In order to confirm these preliminary results further studies are necessary.

## References

[1] Johnson CY, Sharp L, Cotton SC, Harris CA, Gray NM, Little J: Human papillomavirus infection and anxiety: analyses in women with low-grade cervical cytological abnormalities unaware of their infection status. PLoS One. 2011, 6 (6): e21046-10.1371/journal.pone.0021046. 21698168 10.1371/journal.pone.0021046 PMC3116883

[2] Johnson AM, Mercer CH, Beddows S, de Silva N, Desai S, Howell-Jones R, Carder C, Sonnenberg P, Fenton KA, Lowndes C: Epidemiology of, and behavioural risk factors for, sexually transmitted human papillomavirus infection in men and women in Britain. Sex Transm Infect. 2012, 88 (3): 212-217. 10.1136/sextrans-2011-050306. 22261135 10.1136/sextrans-2011-050306 PMC3308471

[3] Sorbye SW, Arbyn M, Fismen S, Gutteberg TJ, Mortensen ES: Triage of women with low-grade cervical lesions–HPV mRNA testing versus repeat cytology. PLoS One. 2011, 6 (8): e24083-10.1371/journal.pone.0024083. 21918682 10.1371/journal.pone.0024083 PMC3168878

[4] Wright TC: Cox JT, Massad LS, Twiggs LB, Wilkinson EJ: 2001 Consensus Guidelines for the management of women with cervical cytological abnormalities. JAMA. 2002, 287 (16): 2120-2129. 10.1001/jama.287.16.2120. 11966387 10.1001/jama.287.16.2120

[5] Zanotti KM, Belinson J: Update on the diagnosis and treatment of human papillomavirus infection. Cleve Clin J Med. 2002, 69 (12): 948-10.3949/ccjm.69.12.948. 951–945, 956 passim 12546268 10.3949/ccjm.69.12.948

[6] Chan JK, Monk BJ, Brewer C, Keefe KA, Osann K, McMeekin S, Rose GS, Youssef M, Wilczynski SP, Meyskens FL: HPV infection and number of lifetime sexual partners are strong predictors for 'natural' regression of CIN 2 and 3. Br J Cancer. 2003, 89 (6): 1062-1066. 10.1038/sj.bjc.6601196. 12966426 10.1038/sj.bjc.6601196 PMC2376964

[7] Hubner NO, Kramer A: Review on the efficacy, safety and clinical applications of polihexanide, a modern wound antiseptic. Skin Pharmacol Physiol. 2010, 23 (Suppl): 17-27. 20829658 10.1159/000318264

[8] Valluri S, Fleming TP, Laycock KA, Tarle IS, Goldberg MA, Garcia-Ferrer FJ, Essary LR, Pepose JS: In vitro and in vivo effects of polyhexamethylene biguanide against herpes simplex virus infection. Cornea. 1997, 16 (5): 556-559. 9294689

[9] Passic SR, Ferguson ML, Catalone BJ, Kish-Catalone T, Kholodovych V, Zhu W, Welsh W, Rando R, Howett MK, Wigdahl B: Structure-activity relationships of polybiguanides with activity against human immunodeficiency virus type 1. Biomed Pharmacother. 2010, 64 (10): 723-732. 10.1016/j.biopha.2010.10.001. 21106331 10.1016/j.biopha.2010.10.001 PMC3776307

[10] Solomon D, Davey D, Kurman R, Moriarty A, O'Connor D, Prey M, Raab S, Sherman M, Wilbur D, Wright T: The 2001 Bethesda System: terminology for reporting results of cervical cytology. JAMA. 2002, 287 (16): 2114-2119. 10.1001/jama.287.16.2114. 11966386 10.1001/jama.287.16.2114

[11] Sellors JW, Sankaranarayanan R: Colposcopy and Treatment of Cervical Intraepithelial Neoplasia. 2003, World Health Organization: A Beginners' Manual

[12] Munoz N, Castellsague X, de Gonzalez AB, Gissmann L: Chapter 1: HPV in the etiology of human cancer. Vaccine. 2006, 24 (3): 1-10. 16949995 10.1016/j.vaccine.2006.05.115

[13] Ho GY, Bierman R, Beardsley L, Chang CJ, Burk RD: Natural history of cervicovaginal papillomavirus infection in young women. N Engl J Med. 1998, 338 (7): 423-428. 10.1056/NEJM199802123380703. 9459645 10.1056/NEJM199802123380703

[14] Krebs FC, Miller SR, Ferguson ML, Labib M, Rando RF, Wigdahl B: Polybiguanides, particularly polyethylene hexamethylene biguanide, have activity against human immunodeficiency virus type 1. Biomed Pharmacother. 2005, 59 (8): 438-445. 10.1016/j.biopha.2005.07.007. 16154720 10.1016/j.biopha.2005.07.007

[15] Marelli G, Papaleo E, Origoni M, Caputo L, Ferrari A: Polyhexamethylene biguanide for treatment of external genital warts: a prospective, double-blind, randomized study. Eur Rev Med Pharmacol Sci. 2005, 9 (6): 369-372. 16479742

[16] Werthen M, Davoudi M, Sonesson A, Nitsche DP, Morgelin M, Blom K, Schmidtchen A: Pseudomonas aeruginosa-induced infection and degradation of human wound fluid and skin proteins ex vivo are eradicated by a synthetic cationic polymer. J Antimicrob Chemother. 2004, 54 (4): 772-779. 10.1093/jac/dkh407. 15355938 10.1093/jac/dkh407

[17] Fabry W, Trampenau C, Bettag C, Handschin AE, Lettgen B, Huber FX, Hillmeier J, Kock HJ: Bacterial decontamination of surgical wounds treated with Lavasept. Int J Hyg Environ Health. 2006, 209 (6): 567-573. 10.1016/j.ijheh.2006.03.008. 16872896 10.1016/j.ijheh.2006.03.008

[18] Yoon CS, Kim KD, Park SN, Cheong SW: alpha(6) Integrin is the main receptor of human papillomavirus type 16 VLP. Biochem Biophys Res Commun. 2001, 283 (3): 668-673. 10.1006/bbrc.2001.4838. 11341777 10.1006/bbrc.2001.4838

[19] Joyce JG, Tung JS, Przysiecki CT, Cook JC, Lehman ED, Sands JA, Jansen KU, Keller PM: The L1 major capsid protein of human papillomavirus type 11 recombinant virus-like particles interacts with heparin and cell-surface glycosaminoglycans on human keratinocytes. J Biol Chem. 1999, 274 (9): 5810-5822. 10.1074/jbc.274.9.5810. 10026203 10.1074/jbc.274.9.5810

[20] Aplin AE, Howe A, Alahari SK, Juliano RL: Signal transduction and signal modulation by cell adhesion receptors: the role of integrins, cadherins, immunoglobulin-cell adhesion molecules, and selectins. Pharmacol Rev. 1998, 50 (2): 197-263. 9647866

[21] Van der Flier A, Sonnenberg A: Function and interactions of integrins. Cell Tissue Res. 2001, 305 (3): 285-298. 10.1007/s004410100417. 11572082 10.1007/s004410100417

[22] Gilbert P, Moore LE: Cationic antiseptics: diversity of action under a common epithet. J Appl Microbiol. 2005, 99 (4): 703-715. 10.1111/j.1365-2672.2005.02664.x. 16162221 10.1111/j.1365-2672.2005.02664.x

[23] Kirschberg T, Parrish J: Metal chelators as antiviral agents. Curr Opin Drug Discov Devel. 2007, 10 (4): 460-472. 17659488

[24] Kines RC, Thompson CD, Lowy DR, Schiller JT, Day PM: The initial steps leading to papillomavirus infection occur on the basement membrane prior to cell surface binding. Proc Natl Acad Sci U S A. 2009, 106 (48): 20458-20463. 10.1073/pnas.0908502106. 19920181 10.1073/pnas.0908502106 PMC2787115

[CR25] The pre-publication history for this paper can be accessed here:http://www.biomedcentral.com/1472-6890/12/17/prepub

